# “Spanish flu,” encephalitis lethargica, and COVID‐19: Progress made, lessons learned, and directions for future research

**DOI:** 10.1111/ene.16312

**Published:** 2024-05-14

**Authors:** Michael Brainin, Yvonne Teuschl, Ellen Gelpi

**Affiliations:** ^1^ Department for Clinical Neurosciences and Preventive Medicine Danube University Krems Krems Austria; ^2^ Division of Neuropathology and Neurochemistry, Department of Neurology Medical University of Vienna Vienna Austria

**Keywords:** COVID‐19, encephalitis lethargica, history of neurology, public health

## Abstract

One hundred years ago, an influenza pandemic swept across the globe that coincided with the development of a neurological condition, named "encephalitis lethargica" for the occurrence of its main symptom, the sudden onset of sleepiness that either developed into coma or gradually receded. Between 1917 and 1920, mortality of the flu was >20 million and of encephalitis lethargica approximately 1 million. For lessons to be learned from this pandemic, it makes sense to compare it with the COVID‐19 pandemic, which occurred 100 years later. Biomedical progress had enabled testing, vaccinations, and drug therapies accompanied by public health measures such as social distancing, contact tracing, wearing face masks, and frequent hand washing. From todays' perspective, these public health measures are time honored but not sufficiently proven effective, especially when applied in the context of a vaccination strategy. Also, the protective effects of lockdowns of schools, universities, and other institutions and the restrictions on travel and personal visits to hospitals or old‐age homes are not precisely known. Preparedness is still a demand for a future pandemic. Clinical trials should determine the comparative effectiveness of such public health measures, especially for their use as a combination strategy with vaccination and individual testing of asymptomatic individuals. It is important for neurologists to realize that during a pandemic the treatment possibilities for acute stroke and other neurological emergencies are reduced, which has previously led to an increase of mortality and suffering. To increase preparedness for a future pandemic, neurologists play an important role, as the case load of acute and chronic neurological patients will be higher as well as the needs for rehabilitation. Finally, new chronic forms of postviral disease will likely be added, as was the case for postencephalitic parkinsonism a century ago and now has occurred as long COVID.

## INTRODUCTION

The so‐called “Spanish flu” was the first globally documented viral pandemic and was active mostly between 1917 and 1920 in Europe, the USA, and other countries. It differed from other globally prevalent and active diseases such as polio or measles insofar as its infectious spread was in distinct waves that resulted in a high mortality of children and adults. It killed >20 million people worldwide, more than had died during the first World War [1]. It incapacitated large areas of urban living and led to the closing of schools, universities, and other institutions. Public transportation was affected, as was any kind of public communication. Although the mode of transmission by air was eventually identified as the most likely mode of transmission, protection from being afflicted was not clear when the pandemic hit initially. It brought along a widespread fear of contagion. Wearing of masks, hand hygiene, avoiding public gatherings, and isolation of the diseased in special hospital wards were the main measures of protection for individuals [2]. Approximately 2‐3 million people developed an acute neurological disorder named "encephalitis lethargica," which had a high mortality of approximately 40%, receded only slowly, and in the mid‐1920s had almost disappeared. Late, chronic forms persisted and occurred up to the late 1930s, and severe cases were mostly treated in psychiatric institutions. Some patients also developed a postencephalitic parkinsonism (PEP). In stark contrast to the memorials for the fallen soldiers of the recent war, almost no memorials were set up in remembrance of this deadly pandemic, which speaks of a remarkable repression in the public awareness of this deadly disease (Table 1).

**TABLE 1:** Comparison of Spanish flu and COVID‐19 and recommendations for future action and areas of research.

	Encephalitis lethargica	Spanish flu	COVID‐19	Recommendations and areas for research
Origin	?	?	?^a^	Install global surveillance
Genetic sequence	No	H1N1 virus	SARS‐CoV‐2 virus sequence published within 2 weeks	
Mortality	1 mio	>20 mio	17.2 mio	
Transmission	?	? (airborne)	Aerosol	
Vaccination	No	No	Yes	Enable global vaccination programs, vaccination plus strategy
Advice to wear face masks and personal hygiene (hand washing)	Yes	Yes	Yes	Test efficacy in various settings
Advice to avoid public gatherings	Yes	Yes	Yes	Test efficacy in various settings
"Lockdowns" (closing of schools, institutions, and nonvital shops)	Partially	Partially	Yes	Large interregional differences, global recommendations needed
Pharmacological treatment	No	No	Yes^b^	Install processes for availability and affordability
Social distancing	Yes	Yes	Yes	
Systematic contact tracing (individual and community)	No	No	Yes	Antibodies or PCR for individuals
				Wastewater analysis for contamination for communities and urban regions
Global warning	No	No	Yes	Global readiness and surveillance of potential spillovers
Late clinical manifestations	Postencephalitic parkinsonism^c^	No	Long COVID	Neurological observations (registries) and global treatment networks needed

Abbreviation: PCR, polymerase chain reaction; ?, unknown; mio, Million.

^a^ It has not been established whether the virus originated at a high‐security laboratory in Wuhan, China, or developed spontaneously.

^b^ Remdesivir became available only late in the pandemic, was very costly, and was not globally distributed. Similarly, the combination of Nirmatrelvir and Ritonavir for early treatment was not easily available even in high‐income countries. Other therapies were not available early in the pandemic, and their availability and affordability differed greatly between countries. For a current overview see Agarwal et al. [19].

^c^ In children and juveniles, increased uncontrolled psychomotor activity [11].

Although the flu pandemic is considered to be caused by H1N1 influenza virus of avian origin [3], there is no consensus regarding whether the virus had caused the acute neurological illness or the late manifestations [3, 4, 5] or where it originated from. It is acknowledged that the worldwide spread of the viral infection started during 1915 and reached a peak between 1918 and 1919. It is estimated that 500 million people or one third of the world's population became infected by the virus. The number of deaths was estimated at >20 million [1]. Mortality from Encephalitis lethargica was high in people younger than 5 years, 20–40 years old, and 65 years or older. The high mortality among younger adults, especially those in the 20–40‐year age group, was a unique feature of this pandemic [6].

Neither individual or communal testing to follow the spreading nor a vaccine to protect against the influenza infection had been developed, and no antibiotics were known at that time to treat secondary bacterial infections. Control efforts worldwide were limited to nonpharmacological interventions such as isolation, quarantine, personal hygiene, and use of disinfectants [2].

## FIRST OBSERVATIONS AND NEUROLOGICAL DESCRIPTION OF ENCEPHALITIS LETHARGICA

The first patients with neurological symptoms were seen by Constantin von Economo (1876–1931) at the Neurological‐Psychiatric Clinic at the Allgemeine Krankenhaus in Vienna, Austria [7, 8]. This institution was at that time led by Julius Wagner‐Jauregg (who was to be awarded the Nobel Prize in 1927 for the discovery of malaria therapy for progressive paralysis). Not only was the first clinical description of encephalitis lethargica in 1917 von Economos' achievement, in the same year he published detailed neuroanatomical reports [9]. French neurologists had challenged von Economo on his first description, but von Economo could prove that his publication was prior. Ludo van Bogaert (who was the founder of the World Federation of Neurology in 1957) clearly gave von Economo the credit for the first description in his authoritative biography [10].

In his first cases seen in the winter of 1916 at the psychiatric clinic in Vienna, he observed that their common symptom was a continuous sleep manifestation. These patients had to be awakened for intake of food and drink, after which they fell again into their deep slumber. It turned out that several days before their admission they had suffered from headache, nausea, and shivering. Discrete signs of encephalitis were noted, which did not increase in severity, but patients developed sleepiness. Regularly, ocular muscle paresis was seen mostly in the region of the oculomotor nuclei, most often as conjugate paresis or nystagmus. Also, oculogyric crises frequently occurred. Finally, some cases developed delirium or deep coma. This often led to rapid death. At that time, von Economo had detected mild signs of encephalitis in the pathologic specimen he had investigated. Apart from the most frequent somnolent–ophthalmoplegic form, he separated a hyperkinetic form and an amyostatic–akinetic form resembling acute parkinsonism [7, 8, 9]. In later observations, he summarized that mortality occurred in 40%, residual morbidity in approximately one third, and recovery in another one third of the cases. Patients, once recovered, could develop PEP after a latency of months or years [11]. Acute forms of encephalitis lethargica were seen from 1915 to 1925, with the first cases appearing in Italy [11], and cases of PEP were described up to 1938 [6]. Neuropathological studies performed later in patients with long‐lasting PEP have revealed a neurodegenerative condition characterized by neuronal loss preferentially in subcortical regions including the substantia nigra and basal ganglia, associated with a neuronal and glial accumulation of tau protein, reminiscent of progressive supranuclear palsy but with different molecular composition [12, 13, 14].

The etiology of encephalitis lethargica and PEP is still debated, although Von Economo surmised that the nasal mucosa may predispose to increasing permeability of the "encephalitic virus" [7, 8]. So far, no definite etiologic finding has been reported, and it is unclear whether it was caused by the influenza virus itself, other environmental factors, or (postinfectious) autoimmune mechanisms by molecular mimicry [15]. Studies in serology, reverse transcriptase polymerase chain reaction, immunohistochemistry, and recent next generation sequencing studies have been negative for viral agents [12].

Patients with postencephalitic parkinsonism included the symptoms bradykinesia, rigidity, akinesia, amimia, postural instability, gait disturbance with falls, resting tremor, and paradoxical kinesia. Furthermore, among chronic cases, oculogyric crises and psychiatric manifestations were frequent. A summary of these observations was published by von Economo in 1929 in a monograph [11]. More recently, Oliver Sacks reported his observations as a neurologist caring for such chronic cases in a psychiatric institution in New York, and that they initially showed a good response to levodopa but this effect was wearing off quite quickly [16]. A dramatized version of these observations has been shown in the Oscar‐winning movie *Awakenings* (1990) with Robert de Niro.

In hindsight, it seems of importance to look at the interpretations of von Economo for the late manifestations as seen from his pathological studies. He describes an abulic disorder (a basic disorder of volition; patients are unable to develop a plan for a willed action) in contrast to an akinetic disorder (patients are able to develop a plan for a voluntary action but are unable to execute this action) [11]. This might be of relevance in the ongoing discussion about a disease model for postviral fatigue states.

## DEVELOPMENT AND SYMPTOMS OF COVID‐19

Comparing the progress in biomedicine and public health measures with the recent COVID‐19 pandemic, a number of conclusions can be drawn for future research and the development of improved preparedness for future pandemics. It must be considered, however, that many questions regarding COVID‐19 are still unanswered, and relevant research is still ongoing. Therefore, COVID‐19 is considered a moving target. Nevertheless, many publications are already available today, among them the most comprehensive and authoritative Lancet Commission Report on the lessons for the future from the COVID‐19 pandemic [17]. With this report, the merit of some measures to be advised and research strategy to be developed can be evaluated.

First, some similarities between Spanish flu or the 1915–1930 influenza pandemic and the recent COVID‐19 pandemic are obvious. There are also clear differences, such as in clinical symptoms, the severity of illness in COVID‐19 being greater in older people, with almost no acute symptoms occurring in children. Nevertheless, the intensity at which both pandemics afflicted entire populations and its likely modes of prevention are comparable.

The milestones in the discovery and management of COVID‐19 also contrast against the pandemic occurring 100 years earlier. Whereas the identification of the virus SARS‐CoV‐2 and its dangerous spread of infection was done in a matter of days, for the Spanish flu pandemic it took a long time. The virus was never identified, and the mode of propagation (airborne, contact, or otherwise) took a long time to identify. Nothing describes better the progress in biomedicine and pandemic handling than these factors 100 years apart.

Differences between the pandemics concerning neurological manifestations are relevant. COVID‐19 is not primarily a neurological disorder. Its major symptoms as seen from its first outbreaks were pulmonary and only secondarily did neurological manifestations occur. The early loss of smell and taste has been associated with nervous involvement, as have been peripheral neuropathies or states of encephalopathy. Other conditions, like stroke, have been related to a hypercoagulative state. But as in encephalitis lethargica, no clear proof has yet been provided that SARS‐CoV‐2 is a bona fide neurotropic virus and most reports provide hints for systemic and/or immune‐mediated mechanisms related to the development of acute/subacute neurological symptoms. Whether some of the patients with or without post‐COVID syndrome will develop a chronic neurological/neurodegenerative condition like PEP after encephalitis lethargica cannot be foreseen yet, but should be closely monitored. Although 80% of SARS‐CoV‐2‐infected patients experience mild symptoms, a significant proportion can be critically ill, including those who are older and those with comorbidities. Upper respiratory symptoms and fever remain the most common presenting symptoms. Extrapulmonary complications include cardiovascular, neurological, or gastrointestinal systems [18].

What were the first observations and how were they handled and communicated? It is well known that the first report came from Wuhan, China about an outbreak of a viral epidemic, which reached the World Health Organization (WHO) on 31 December 2019. It was not clear where exactly this virus had originated. It is believed either to have evolved spontaneously or to have come from a virus laboratory in Wuhan. The genomic sequence was readily made available by Chinese virologists, who identified a SARS‐CoV as the agent in early January. In Wuhan and five other regions, a lockdown was declared on 23 January 2020; the WHO declared the novel coronavirus a global emergency 1 week later on 30 January (all data from Sacks et al. [17]).

COVID‐19 went global rapidly. On 24 January, the first case was seen in Europe, and of the first 47 cases diagnosed in Europe, 14 patients had recently visited China. After a long wait of 4 months after the outbreak, on 5 June, the WHO recommended the use of face masks in public. Until then, it had advised only to restrict cases in medical settings.

Clinically, there was a rapid onset with influenzalike symptoms that often deteriorated to pulmonary insufficiency and afforded intensive care including artificial ventilation. Neurological symptoms included disorders of consciousness, especially in severe cases, ranging from drowsiness to deep coma. Mortality was high, especially in cases that needed ventilation and in elderly or multimorbid persons. Recovery usually was slow, and surviving patients experienced a longer period of muscular weakness and fatigue. According to Sachs et al. there were 6.9 million deaths by the end of May 2022, with an estimated true death toll of 17.2 million [17].

With considerable speed, vaccinations became available within 10 months after the identification of the virus, reflecting an ongoing and responsible partnership between industry and biomedical research but also with health authorities and international health agencies. Richard Horton [20] notes about this extraordinary global collaboration, “…from the sequencing of the virus by Chinese scientists, to the development of the vaccine by immigrants of Turkish heritage in Germany, to its manufactures in Belgium.” He mentions the British efforts by Oxford scientists together with AstraZeneca and concludes that “the lesson of vaccine research is that the sum of scientific cooperation between nations produces greater success than can be achieved by any one group in any country working alone.” This "internationalism" clearly marks a difference and progress from the influenza pandemic of 1915 and encephalitis lethargica, which occurred at a time when no global health organization or even regular international communication was available.

The vaccinations reached large populations. In the USA 64% of the population was vaccinated by 22 January, and in the European Union 71% were fully vaccinated, whereas in Africa the corresponding rate was 10%. In Nigeria, it was only 2%. In total, four waves swept over the globe with differing regional spread and intensity. The WHO recommended that 40% of the world population should be vaccinated to achieve effects from immunity. Vaccinations were also modified according to new upcoming variants (data from Sacks et al. [17]).

These variants occurred over the first 2 years of this pandemic and led to different reactions from governments and health authorities, ranging from single to repeated total lockdowns with closure of all institutions and shops (except emergency services and other vital services), resulting in "flatten the curve" policies, with the intention to reduce infections numbers and hence acute hospital admissions. It was noticed that lockdowns were often declared when the curve was already flattening. Contact tracing was instituted by regional health authorities, as was the obligatory wearing of face masks in public. As a reaction, digitalization of production, education, and communication was developed, resulting in large numbers of office workers, teachers, students, and pupils relying on digital tools instead of personally attending their office or school.

According to Sachs et al. [17], the positive aspects must also be acknowledged: the private–public partnerships for rapid development of vaccines that saved uncountable lives, the high‐income countries' support to households, businesses, and employers, and the support from the World Bank and International Monetary Fund.

Some weaknesses and failures also became evident. In many countries, a growing distrust in governmental regulations and health care rules developed. With the increasing number of lockdowns, the measures were not followed by all, nor were the calls for a booster vaccination. Some noted failures included the lack of timely notification of the outbreak, costly delays in acknowledging the crucial airborne exposure pathway, lack of coordination between countries regarding containment strategies, failure of governments to examine and adopt best evidence for controlling the virus, shortfall of funding for low‐ and middle‐income countries, failure to ensure adequate global supplies, lack of data, and inability and unwillingness to combat infection.

These "failures" must be seen in the light of the WHO having previously developed an emergency plan for pandemic handling for governments. Unfortunately, this plan was not recognized as a priority. In particular the recommendation for setting aside a budget and exercising an annual alarm at the national level was not followed, which enabled the upcoming pandemic to come as a surprise for practically all governments. A detailed report of the WHO compiled during the pandemic in 2021 notes the need for prioritized preparatory action [1].

## WHAT CAN WE LEARN FROM BOTH PANDEMICS?

First, we realize that there was a century of biomedical progress between the pandemics. We can admire the enormous progress of science and biomedicine achieved in the past 100 years. This progress is seen in the fields of genetics, virology, vaccination research, therapy development, and public health measures. Whereas with Spanish flu and the appearance of encephalitis lethargica there was no clear idea which virus was involved (and this is still unclear for encephalitis lethargica), the public was informed at best at the national level, the mode of transmission was not proven, and therapy was limited to passive care and palliative measures. One hundred years later, a worldwide regulating health body, the WHO, had been founded and was globally active. Consequently, there were existing international communications, surveillance of regional epidemics, and output of global recommendations and warnings. Biomedical progress had been able to provide a rapid reaction to identify a global virus threat and further to develop a vaccination strategy to mitigate its spread and its deleterious effects. Eventually, and with hitherto unknown speed, medications were licensed and vaccinations developed.

Intensive care and artificial ventilation were useful for many patients; its shortages and reduced availability became visible during the height of the pandemic, resulting in a selection process for accepting patients in need of such services. This led to an increase in mortality in many regions and urban centers that could have been avoided if timely preparations and early provisions were taken. An overall experience also was the reluctance of many people to undergo vaccinations for fear of dominating side effects. This reluctance sometimes culminated into open and irrational denial of the pandemic and its consequences [20, 21].

Overburdening of hospital capacities had also led to a decrease of management capacities for non‐COVID emergencies. This has been noted in many countries, especially for acute strokes and cardiovascular events [22]. Stroke units have been utilized for COVID care, and their capacity for treating strokes was reduced. Moreover, medical capacities for chronic and oncological patients were downsized. Postponement or cancellation of treatments had deleterious effects. Rehabilitation has suffered as well. For neurological patients, timely rehabilitation is essential for optimal recovery. At the same time, telerehabilitation was boosted and has been shown to be highly efficient, popular among patients, and cost‐effective. To rely on traditional hospital settings is not enough. In a modelling study from England, it has been shown that even with an increase in hospital capacity of 30% several years would be needed for the backlog to clear [23]. Overall, the COVID‐19 pandemic has had a devastating physical and mental impact on the millions of people living with noncommunicable diseases. People of older age and those living with the consequences of a stroke or other chronic neurological/neurodegenerative condition or with cardiovascular diseases, obesity, diabetes, kidney disease, or hypertension are at a particularly greater risk. The Global Coalition for Circulatory Health calls for increased support for health care workers, global vaccine equity, and embracing new models of care and digital health solutions, as well as fiscal policies on unhealthy commodities to support these investments [24]. A statement of the World Stroke Organization reads that not only do stroke patients appear to be more susceptible to severe infection, but the pandemic is having major implications on how we deliver stroke care while ensuring the safety of both our patients and health care professionals. COVID‐19 infection itself has also been described as a risk factor for stroke. The World Stroke Organization has been monitoring the impact of the pandemic globally and has identified an initial marked fall in stroke presentations as well as a widespread impact on stroke services [25].

One of the most important lessons learned from both pandemics is that constant surveillance is needed on national and international levels. It was noted that the WHO should not only declare a state of emergency but also recommendations for handling the situation [13].

Preparedness for a new pandemic is vital for future management. To observe regional infections and their spread leads to estimates of spillover of infections and increased risk outside the region. It is essential that not only the occurrence but also the type and intensity of the response needed to any "public health emergency of international concern" are also communicated. This includes emergencies such as monkeypox, Zika, Ebola, and poliomyelitis. Also, countermeasure strategies for either eradication, suppression, or mitigation should be included when declaring such an emergency [17].

The scientific evidence for most public health measures is still weak. It is discouraging to see medical therapies had been studied almost exclusively with proper methods and with cohorts large enough but hardly any general measures. According to Sachs et al., there had been 2465 drug trials and 109 vaccine trials during the pandemic but only 16 trials of preventive intervention and two randomized clinical trials on the effectiveness of wearing face masks [17]. Moreover, strategies for monitoring long‐term neurological disabilities should be planned.

Despite great advances in biomedical research and in international health surveillance, both pandemics show persistent uncertainties that need to be recognized. First, a state of preparedness needs to be heightened and kept up. Second, a set of measures has to be considered by health authorities and governments according to evidence still to be provided. Third, strategies of research must focus on public health measures and their efficacy, and placement within other strategies such as vaccination strategies and strategies for testing of asymptomatic individuals should be studied. It may well be that one measure taken at one time is extremely effective but when taken at a later point in time hardly shows an effect. Finally, neurologists seem to play a vital role in such pandemics, because of the potential acute involvement of the nervous system, which controls all vital functions and thus involves the nervous system as end organ, but also because of late manifestations that need to be recognized and treated [26].

## AUTHOR CONTRIBUTIONS

**Michael Brainin:** Conceptualization; writing – original draft; investigation. **Yvonne Teuschl:** Writing – review and editing. **Ellen Gelpi:** Writing – review and editing.

## CONFLICT OF INTEREST STATEMENT

None of the authors has any conflict of interest to disclose.

## Data Availability

Data sharing not applicable to this article as no datasets were generated or analysed during the current study.
